# Determinants of Participation in a Web-Based Health Risk Assessment and Consequences for Health Promotion Programs

**DOI:** 10.2196/jmir.2387

**Published:** 2013-08-09

**Authors:** Maurice AJ Niessen, Eva L Laan, Suzan JW Robroek, Marie-Louise Essink-Bot, Niels Peek, Roderik A Kraaijenhagen, Coen K Van Kalken, Alex Burdorf

**Affiliations:** ^1^NIPED Research FoundationAmsterdamNetherlands; ^2^Department of Public HealthAcademic Medical Center, University of AmsterdamAmsterdamNetherlands; ^3^Department of Public HealthErasmus MCRotterdamNetherlands; ^4^Department of Medical InformaticsAcademic Medical CenterAmsterdamNetherlands

**Keywords:** participation, Internet, workplace, health promotion, health risk assessment, reach

## Abstract

**Background:**

The health risk assessment (HRA) is a type of health promotion program frequently offered at the workplace. Insight into the underlying determinants of participation is needed to evaluate and implement these interventions.

**Objective:**

To analyze whether individual characteristics including demographics, health behavior, self-rated health, and work-related factors are associated with participation and nonparticipation in a Web-based HRA.

**Methods:**

Determinants of participation and nonparticipation were investigated in a cross-sectional study among individuals employed at five Dutch organizations. Multivariate logistic regression was performed to identify determinants of participation and nonparticipation in the HRA after controlling for organization and all other variables.

**Results:**

Of the 8431 employees who were invited, 31.9% (2686/8431) enrolled in the HRA. The online questionnaire was completed by 27.2% (1564/5745) of the nonparticipants. Determinants of participation were some periods of stress at home or work in the preceding year (OR 1.62, 95% CI 1.08-2.42), a decreasing number of weekdays on which at least 30 minutes were spent on moderate to vigorous physical activity (OR_*dayPA*_0.84, 95% CI 0.79-0.90), and increasing alcohol consumption. Determinants of nonparticipation were less-than-positive self-rated health (poor/very poor vs very good, OR 0.25, 95% CI 0.08-0.81) and tobacco use (at least weekly vs none, OR 0.65, 95% CI 0.46-0.90).

**Conclusions:**

This study showed that with regard to isolated health behaviors (insufficient physical activity, excess alcohol consumption, and stress), those who could benefit most from the HRA were more likely to participate. However, tobacco users and those who rated their overall health as less than positive were less likely to participate. A strong communication strategy, with recruitment messages that take reasons for nonparticipation into account, could prove to be an essential tool for organizations trying to reach employees who are less likely to participate.

## Introduction

Seven modifiable risk factors account for more than half of the chronic disease burden: high blood pressure, tobacco use, excess alcohol consumption, high serum cholesterol, overweight, low fruit/vegetable intake, and physical inactivity [1]. The workplace is considered to be an excellent setting for health promotion programs that target these risk factors, not only because a large proportion of the population can be reached, but also because it makes use of a natural social network and can facilitate the creation of a health-conscious environment [2-4]. Web-based interventions serve as a feasible and acceptable delivery method for these programs because they can provide scale at a relatively low cost per employee [5,6]. In addition, Internet access is available 24 hours a day, 7 days a week, which may serve both the employer and the employee, as program access is available across work shifts and into vacation and leisure time [6].

Recent reviews of effectiveness studies concluded there is sufficient evidence that worksite health promotion programs (WHPPs) have meaningful effects on a number of risk factors [7,8]. The latter is directly beneficial for the employer: implementing a WHPP can lead to reductions in both absenteeism and productivity loss at work [9,10]. However, a lack of employee participation presents an important barrier to the impact of WHPPs [7,11]. Since most intervention studies on WHPPs randomize workers who have agreed to participate in the studies, it is largely unknown whether those who could benefit most from the intervention are as likely to participate as those who may have already been making more healthful choices [12,13]. The importance of studying determinants of participation in WHPPs was already emphasized 25 years ago and has been underscored ever since [14-16]. Still, in 2009, the authors of a review concluded that few studies have evaluated the influence of health, lifestyle, and work-related factors on participation, which hampers insight into the underlying determinants of participation in WHPPs, and ultimately, the influence of selective participation on the effectiveness of these WHPPs [3]. Except for the finding that women enroll more often than men, no consistent determinants of participation in WHPPs aimed at physical activity and nutrition were found [3].

With regard to Web-based delivery of WHPPs, it has been reported that women and older people are more likely to enroll in these programs, as they more often use the Internet for searching for health-related information. It has also been postulated that individuals with a low educational level are less likely to use Web-based WHPPs, as those with less formal education are less likely to continue the adoption of innovations [17].

One type of WHPP that is frequently offered is the health risk assessment (HRA), which screens for risk factors for chronic diseases [7,10] and delivers verbal or written feedback on one’s personal risk profile along with subsequent recommendations for lifestyle improvements. While an HRA is often used as a gateway intervention to broader WHPPs, it can also be utilized as a tool for stimulating the initiation of health behavior change [4,7]. In the current study, our aim was to analyze whether individual characteristics (including demographics, health behavior, self-rated health, and work-related factors) are associated with participation and nonparticipation in a Web-based HRA [9] implemented among employees in the Netherlands.

## Methods

### Participating Organizations and Study Design

In this cross-sectional study, the HRA was implemented in five Dutch organizations, which included a university medical center, a large state-owned bank, a small bank, a financial institution, and the Dutch branch of an American multinational technology and consulting corporation. The HRA was applied in a pilot study among selected departments of the university medical center, which employed over 10,000 employees in 2009. The large state-owned bank was nationalized as a result of the global financial crises and employed more than 27,000 employees in 2009. Starting in 2006, its employees were gradually invited to enroll in the HRA. Renewed enrollment in the HRA was offered to employees 3 years after the first HRA was completed. In the current study, we included all invitees from 2009 who had not previously participated in the HRA. All workers from the small bank (<1000 employees) were invited, and from the financial institution (>3000 employees), all invitees from 2009 who had not previously participated in the HRA (renewed participation offered after 3 years) were included in this study. The Dutch branch of the American multinational technology and consulting corporation employed over 4500 employees in 2010. The HRA has been implemented in the organization since 2006. Two years after initial participation, renewed enrollment in the HRA is offered. In this study, we included all employees who were invited during the first and second quarters of 2010 and had not previously participated in the HRA.

### Procedures

Employees were invited to participate in the HRA during the period from January 2009 to August 2010. The university medical center imposed an age criterion, inviting employees who were at least 45 years old. Upper management encouraged managers of selected departments to stimulate enrollment in the HRA among their workers. The HRA was also highlighted in the in-house employee magazine.

During the study period, invitations to participate in the HRA were sent by the human resources department, management, or the safety, health, and welfare services of the organizations involved. The invitation email included a description of the HRA and informed employees that participation was voluntary and free of charge, that all personal data would be treated confidentially, and that no individual results would be shared with their employer or any other party. No incentives were offered.

The HRA is called “The Prevention Compass” [4,9]. In the assessment phase, a Web-based health questionnaire is completed (in 30-45 minutes), biometric measurements (height, weight, waist circumference, blood pressure) are taken, and blood, urine, and feces samples are analyzed. A personalized Web-based health report and health plan is automatically generated only after all health data are collected. At this point, the HRA is completed.

Employees were defined as enrollees when they enrolled in the program by activating their online account during the inclusion period. This period varied (3-12 months), as larger organizations chose to invite their employees gradually. Enrollees who completed all HRA measurements within 1 year after the inclusion period had ended were classified as participants. Those who enrolled but did not complete all measurements were labeled dropouts. Employees who had not enrolled in the program after the inclusion period had ended were labeled nonparticipants. The provider of the HRA sent nonparticipants an email inviting them to complete an online questionnaire. Those who responded to the online questionnaire were classified as responders, and those who did not respond were labeled nonresponders. Informed consent was obtained from all study participants prior to the study in accordance with the requirements for identifiable data collection in the Dutch Code of Conduct for Observational Research.

### Measurements

For all study participants, gender and date of birth were available from the HRA invitation lists used by the organizations involved. Other individual characteristics (which included educational level, self-rated health, physical activity, body mass index (BMI), alcohol consumption, stress, work ability, and absenteeism during the previous year) were collected from the Web-based health questionnaire component of the HRA as part of a larger set of health data collected to generate a personal health report. As nonparticipants did not participate in the HRA and its Web-based health questionnaire, an online questionnaire was created that was made up almost entirely of the questions related to the above-mentioned individual characteristics of this study. Our goal was to lower the threshold and make it easier for nonparticipants to complete the questionnaire. Therefore, it was anonymous, no account had to be activated, and it took 10 minutes to complete. The questions relating to the individual characteristics were identical for participants and nonparticipants.

To determine educational level, respondents were asked to check 1 of 9 categories (ranging from no education to doctorate level) that indicated the highest level of education ever completed. Self-rated health [18,19] was measured by one question: “How do you rate your health in general?” The response options were “very good”, “good”, “moderate”, “bad”, or “very bad”. Because of a lack of observations for the option “very bad”, this category was merged with “bad” prior to the regression analysis.

One item derived from the Dutch version of the International Physical Activity Questionnaire [20] was used to assess the number of weekdays on which at least 30 minutes were spent on moderate to vigorous physical activity. BMI was based on height and weight as reported by respondents on the online questionnaire (nonparticipants) or measured by trained personnel (participants), and categorized into normal weight (BMI<25 kg/m^2^), overweight (25≤BMI<30 kg/m^2^), or obese (BMI≥30 kg/m^2^).

Alcohol consumption was measured in units of alcohol per week based on a standard alcohol questionnaire of the Dutch Municipal Health Service (“GGD Monitor”). Because few participants reported high levels of alcohol consumption, answer categories “29–35 units”, “36–42 units”, “43–50 units”, and “> 50 units” were merged with “22–28 units” into “≥22 units.” One item measured the frequency of tobacco use (none, occasionally, weekly, or daily). Answer categories “daily” and “weekly” were merged into “daily/weekly” as a measure of frequent tobacco use.

Items from the INTERHEART study were used to measure general and financial stress [21]. In accordance with the methods used in that study, 2 items relating to stress at home and stress at work were combined into a general stress scale and graded as follows: (1) never experienced stress, (2) experienced some periods at home or at work, (3) experienced several periods at home or at work, or (4) experienced permanent stress at home or at work. Level of financial stress was defined as (1) little or none, (2) moderate, or (3) high or severe.

Work ability was measured with the single-item question on work ability from the Work Ability Index (WAI) [22]. Both the WAI and the single-item question show similar patterns of associations with absenteeism, health, and symptoms [23]. On the single-item question, respondents were asked to assess their current work ability compared with their lifetime best, with a possible score of 0 (“completely unable to work”) to 10 (“work ability at its best”).

Absenteeism during the previous 12-month period was determined by a question that classified the number of absenteeism (calendar) days related to health problems into 1 of 5 categories (0, 1-9, 10-24, 25-99, 100-365) [24].

### Statistical Analysis

Means and standard deviations were presented for the continuous variables of age, physical activity, and work ability. Percentages were presented for the dichotomous variable gender and the categorical variables of education, BMI, alcohol consumption, tobacco use, stress at home or work, financial stress, self-rated health, and absenteeism. Enrollees, participants, nonparticipants, questionnaire responders, and nonresponders were compared using the unpaired *t* test for continuous variables and the chi-square test for dichotomous and categorical variables.

Spearman rank correlation coefficients were computed to investigate interrelationships among individual characteristics. Using the Bonferroni approach to control for Type 1 errors across the 132 correlations of the 12 variables, a *P* value of less than .0004 (.05/132=.0004) was required for significance [25]. Correlations had to be at least 0.20 to be considered practically relevant.

Multivariate logistic regression analysis was performed to identify individual characteristics that contributed to participation in the HRA, after controlling for company and all other variables. This method presumes that all individual characteristics are measured for all cases and incomplete cases are discarded, which may result in biased estimates [26]. Therefore, multiple imputation of missing values of independent variables was employed. In multiple imputation, missing data are imputed based on variables correlated with the missing data and causes of missingness. In this study, ordinary least-squares regression models were applied to predict the missing values of continuous and ordinal variables, and discriminant prediction models were applied to the missing values of nominal variables. All individual characteristics as well as participant status (participant vs nonparticipant) were used as covariates in the predictive models. Uncertainty was accounted for by creating 10 imputed datasets [27]. Multivariate logistic regression analysis was carried out on each imputed dataset, producing multiple analysis results. These analysis results were combined using rules established by Rubin [27] to produce one overall analysis, which is reported and compared with the results of complete case analysis.

The SOLAS 4 statistical package was used for the multiple imputation of the missing values. All other analyses were performed using SPSS for Windows, version 19.

## Results

**Figure 1:**
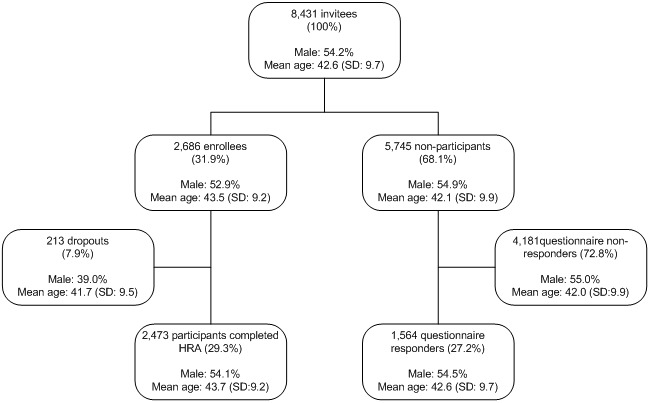
Study flow chart.

**Figure 2:**
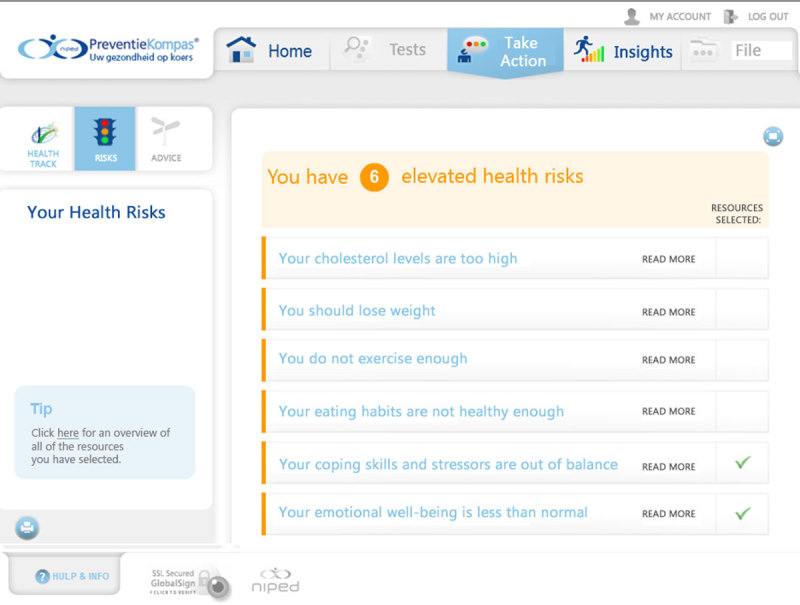
Screenshot of the personal health risk profile page.

The study flow chart is presented in Figure 1. During the study period, 8431 employees were invited to participate in the HRA. Average participation was 31.9% (2686/8431) and ranged from 14.9% to 51.7%: university medical center: 51.7% (206/503), state-owned bank: 29.9% (1282/4284), small bank: 41.0% (213/520), financial institution: 34.3% (824/2404), Dutch branch of American multinational technology and consulting corporation: 14.9% (107/720). The online questionnaire was completed by 27.2% (1564/5745) of the nonparticipants. Data on gender and age were available for 99.5% (8390/8431) of all HRA invitees from the invitation lists. Both enrollees (*P*<.001) and questionnaire responders (*P*=.02) were slightly older compared with nonparticipants and nonresponders. Also, enrollees were less often male (*P*=.046). Of those who enrolled in the HRA, 7.9% (213/2686) did not complete participation (dropouts). Compared with participants who completed the HRA, dropouts were younger (*P*=.002) and less often male (*P*<.001). Dropouts were excluded from further analysis, as no additional data beyond age and gender were available for this group. An example of a personal health risk profile page that was presented to those who completed the HRA is shown in Figure 2.

Table 1 depicts the baseline characteristics of participants (those who completed the HRA) and nonparticipants who filled in the online questionnaire (hereafter described as nonparticipants). Participants were slightly older than nonparticipants. No differences in gender or education were found. Participants engaged in physical activity less frequently, had higher weekly alcohol consumption, and reported having had periods of stress at home or work during the previous year more often. Nonparticipants had lower self-rated health, used more tobacco, and reported slightly lower work ability, a higher level of financial stress, and more absenteeism in the preceding year.

A correlation matrix was computed to ascertain associations between the individual characteristics. Male gender was positively related with alcohol consumption (*r=*.33) and age was positively related with BMI (*r=*.21). A negative correlation (*r=*-.28) was found between the amount of stress at home or work and self-estimated work ability. Stress at home or work was positively correlated (*r=*.21) with financial stress. More positive self-rated health was correlated with higher work ability (*r*=.29) and negatively correlated with the amount of absenteeism during the previous 12-month period (*r*=-.22).

In Table 2, the independent influence of demographics, health behavior, self-rated health, and work-related factors on HRA participation is shown for the imputed datasets (combined results), after controlling for organization (not shown) and all other independent variables. In the multivariate logistic regression analysis model, no effects were found for demographics. Less frequent physical activity, higher weekly alcohol consumption, and some periods of stress at home or work during the previous year remained statistically significantly associated with higher participation. It was also confirmed that less-than-positive self-rated health and tobacco use are significantly associated with lower participation. Higher levels of financial stress, more absenteeism, and lower work ability were no longer significantly related to lower participation.

Complete case analysis confirmed the direction of the reported results based on the imputed datasets. In addition, the following associations attained significance in the complete case analysis. Severe levels of financial stress, good self-rated health, and absenteeism (1-9 days and 100-365 days) were associated with lower participation. Having had several periods of stress at home or work and female gender were associated with higher participation. Also, in the complete case analysis, the association between occasional tobacco use and lower participation was marginally significant (*P*=.06).

**Table 1:** Baseline characteristics of HRA participants and nonparticipants who completed the online questionnaire.

Characteristics		HRA participants N=2473	HRA nonparticipants who completed questionnaire N=1564	*P* value
**Age**		n=2473	n=1564	.001
	Mean (SD)	43.7 (9.2)	42.6 (9.7)	
**Gender, n (%)**		n=2472	n=1564	.81
	Male	1337 (54.1)	852 (54.5)	
	Female	1135 (45.9)	712 (45.5)	
**Education** ^a^, **n (%)**		n=2451	n=1549	.41
	Low	400 (16.3)	266 (17.2)	
	Intermediate	782 (31.9)	464 (30.0)	
	High	1269 (51.8)	819 (52.9)	
**Physical activity**		n=2473	n=1403	<.001
	Weekdays (0-7) ≥30 min., mean (SD)	3.2 (2.1)	3.8 (2.2)	
**Body mass index (BMI), n (%)**		n=2473	n=1404	.42
	Normal weight: BMI <25kg/m^2^	1078 (43.6)	586 (41.6)	
	Overweight: BMI ≥25 - <30 kg/m^2^	1097 (44.4)	637 (45.3)	
	Obese: BMI ≥ 30 kg/m^2^	298 (12.1)	184 (13.1)	
**Alcohol consumption, n (%)**		n=2473	n=1403	<.001
	<1 units/wk	702 (28.4)	552 (39.3)	
	1-7 units/wk	1037 (41.9)	569 (40.6)	
	8-14 units/wk	479 (19.4)	195 (13.9)	
	15-21 units/wk	173 (7.0)	64 (4.6)	
	≥22 units/wk	82 (3.3)	23 (1.6)	
**Tobacco use, n (%)**		n=2471	n=1251	<.001
	None	1961 (79.4)	889 (71.1)	
	Occasional	115 (4.7)	79 (6.3)	
	At least once/wk	395 (16.0)	283 (22.6)	
**Stress—at home or work, n (%)**		n=2436	n=1374	<.001
	Never	278 (11.4)	194 (14.1)	
	Some periods	1298 (53.3)	628 (45.7)	
	Several periods	822 (33.7)	522 (38.0)	
	Permanent	38 (1.6)	30 (2.2)	
**Stress—financial, n (%)**		n=2432	n=1374	<.001
	Little or none	1872 (77.0)	947 (68.9)	
	Moderate	490 (20.1)	352 (25.6)	
	High or severe	70 (2.9)	75 (5.5)	
**Self-rated health, n (%)**		n=2468	n=1564	<.001
	Very good	438 (17.7)	194 (12.4)	
	Good	1684 (68.2)	1055 (67.5)	
	Moderate	328 (13.3)	272 (17.4)	
	Bad or very bad	18 (0.7)	43 (2.7)	
**Absenteeism, n (%)**		n=2469	n=1374	<.001
	0 days	975 (39.5)	462 (33.6)	
	1-9 days	1194 (48.4)	683 (49.7)	
	10-24 days	183 (7.4)	117 (8.5)	
	25-99 days	86 (3.5)	73 (5.3)	
	100-365 days	31 (1.3)	39 (2.8)	
**Work ability**		n=2466	n=1374	
	Mean (SD)	8.1 (1.4)	8.0 (1.5)	.007

^a^Education: Low-lower general secondary/lower vocational; Intermediate-higher general secondary/pre-university/intermediate vocational; High-higher vocational/university.

**Table 2:** Influence of demographics, health, and work-related factors on HRA participation.

Characteristics		OR^a^	95% CI^b^
Age	10 yr intervals	1.127	0.961 - 1.322
Male gender		0.884	0.661 - 1.181
Education^c^	Low^d^		
	Intermediate	1.203	0.813 - 1.780
	High	0.919	0.618 - 1.365
Physical activity	Days per week ≥30 min.(0-7)	0.843	0.793 - 0.895
Body mass index (BMI)	Normal weight: BMI <25 kg/m^2^ ^d^		
	Overweight: BMI ≥25 - <30 kg/m^2^	0.893	0.674 - 1.185
	Obese: BMI ≥30 kg/m^2^	0.938	0.610 - 1.441
Alcohol consumption	<1 units per week^d^		
	1-7 units per week	1.447	1.074 - 1.949
	8-14 units per week	1.971	1.318 - 2.947
	15-21 units per week	2.224	1.210 - 4.088
	≥22 units per week	3.372	1.317 - 8.632
Tobacco use	None^d^		
	Occasional	0.303	0.186 - 0.494
	At least once a week	0.645	0.461 - 0.903
Stress—home or work	Never^d^		
	Some periods	1.618	1.081 - 2.421
	Several periods	1.467	0.950 - 2.226
	Permanent	1.505	0.534 - 4.240
Stress—financial	Little or none^d^		
	Moderate	0.777	0.571 - 1.056
	High or severe	0.650	0.329 - 1.282
Self-rated health	Very good^d^		
	Good	0.711	0.489 - 1.035
	Moderate	0.567	0.344 - 0.935
	Bad or very bad	0.251	0.077 - 0.812
Absenteeism	0 days^d^		
	1-9 days	0.851	0.642 - 1.128
	10-24 days	0.719	0.442 - 1.172
	25-99 days	0.751	0.390 - 1.446
	100-365 days	0.480	0.177 - 1.302
Work ability	(0-10)	1.014	0.919 - 1.120

^a^OR: odds ratio

^b^CI: confidence interval

^c^Education: Low-lower general secondary/lower vocational; Intermediate-higher general secondary/pre-university/intermediate vocational; High-higher vocational/university.

^d^Reference category

## Discussion

### Principal Results and Comparison With Prior Work

In this study, we evaluated the determinants of participation in a Web-based HRA by comparing participants and nonparticipants with regard to demographics, health behavior, self-rated health, and work-related factors. We found evidence of health-related participation, as workers who were more willing to participate in the HRA engaged in physical activity less frequently, consumed more alcohol, and more frequently experienced some periods of stress at home or work. Nonparticipants rated their overall health less positively and used more tobacco.

Participation in the HRA (31.9%) was similar to the response to the nonparticipant questionnaire (27.2%). The crude analysis pointed towards higher participation among older employees and females. These demographic differences were no longer present in the multivariate analysis. Therefore, the Web-based delivery of the WHPP did not result in selective participation by more highly educated, female, or older employees, which could be explained by the high Internet penetration (94%) in the Netherlands [28]. Although other studies have shown no consistent effect of age on participation [3,15], a meta-analysis performed by Robroek and colleagues (2009) found that women are more likely to participate in WHPPs than men [3]. Also, thus far a number of studies have shown fairly consistently that there is lower participation among employees of lower socioeconomic status [14,15,29-33].

The current study found a strong association between physical activity and HRA participation. The likelihood of participating in the HRA increased as the number of weekdays an employee engaged in physical activity decreased. This result seems to indicate that employees who engage less in physical activity want to know about their state of health, and that those already engaged in frequent physical activity find it less important to participate. However, reports on the influence of physical activity on participation have not been consistent, with some studies pointing towards higher participation in WHPPs among the less physically active [30,34] and other studies indicating higher participation among those with low fitness risk [34] or above-average levels of both habitual activity and physical fitness [35].

Participation in the HRA in our study was also associated with alcohol consumption. Higher weekly alcohol consumption increased the likelihood of participating in the HRA. This finding might be explained by the nonstigmatizing way of addressing alcohol consumption through the Internet. No association between excess alcohol consumption and participation was found in a recent study of a Web-based WHPP [36] or other studies of WHPPs [37].

In the current study, employees who experienced stress at home or at work during the prior year were more likely to participate in the HRA. Two other studies evaluated this association and found similar results [38,39]. These findings suggest that the HRA reaches an important group of workers, as workers under psychological strain are especially vulnerable to absenteeism and disability [40].

We showed that individuals who rated their health as “moderate” or “bad/very bad” were less likely to participate in the HRA. Self-rated health is associated with physical and mental functioning [18]. In the long run, it is a robust predictor of all-cause mortality and morbidity, and mortality in a range of conditions including cardiovascular disease and cancer [18]. A more immediate association between self-rated health and self-reported absenteeism in the preceding year was found in the current study. Because of these associations, the lack of participation among employees with less-than-positive self-rated health could be interpreted as a general indication that less healthy employees are less likely to participate. One possible reason for this could be that these individuals are currently under treatment for a physical or mental condition. Receiving current medical treatment is an important reason for nonparticipation in WHPPs [38] and was found to be related to nonparticipation in this particular HRA [41]. One could argue that participating in a WHPP is less relevant for those receiving treatment. However, WHPPs and especially broad-based HRAs are designed to screen for a range of chronic diseases and health behaviors, and these programs are likely to benefit individuals who are already receiving medical treatment in other, potentially isolated, areas of health care. Moreover, not everyone with negative self-rated health is receiving medical care. Another reason for lower participation among employees with lower self-rated health could be less healthy employees’ desire to keep their private life and their work life separate. One study found indications that employees with unhealthy lifestyles or who are in poor health are more likely to resist employer interference with employee health [42]. Lower participation among employees with negative self-rated health has been reported in an earlier study on this HRA [41] and other WHPPs [14], but these reports are not consistent [43].

Our study adds to the fairly consistent reports that tobacco users are less likely to participate in WHPPs [30,33,37,38,44]. Most tobacco users are well aware of their habit’s adverse effects and may find they can foresee the outcome and recommendations if they participate in a WHPP. They may find the prospect of such recommendations patronizing and are probably already being confronted with the negative reactions of others in the workplace or at home as a result of their habit. In the HRA under investigation, tobacco users are not encouraged to feel “guilty” or otherwise “pressured” to quit. Intrinsic motivation is recognized as a necessary ingredient for lasting behavior change. Their freedom of choice is affirmed: he or she is respectfully informed of the health benefits of smoking less or quitting and offered resources for bolstering resolve and self-confidence to become smoke-free. However, it is unlikely that the nonjudgmental aspect of this program was communicated to employees prior to their decision of whether or not to participate in the HRA.

This is the second study to evaluate participant characteristics of the HRA, the Prevention Compass. Our study, conducted with a new cohort, addressed two major limitations of the earlier study, which was reported on in 2011 [41]. First, in the 2011 study, only 14% of the nonparticipants completed the online questionnaire, which formed the basis for the comparison between nonparticipants and participants. As a result, selection bias could have influenced the findings reported in that study. This is hinted at by the substantial difference in reported age between questionnaire responders and nonresponders. Second, we used multivariate analysis in our study. This has the obvious advantage of being able to control for confounding by all other potential determinants. For example, in the 2011 study, it was reported that older employees were likely to participate in the HRA. Also, less self-reported absenteeism was found among participants. We found similar results in the crude analysis of our data. However, in the multivariate analysis, neither age nor absenteeism were still significant determinants. Two of the independent determinants of participation found in the current study—physical activity and alcohol consumption—were not evaluated in the earlier study.

In addition to individual characteristics, program and organizational factors have been linked to participation in WHPPs [37]. Offering financial incentives is one of these factors. Not surprisingly, these incentives increase participation**,** but one can wonder whether such an external motivator helps to bring about lasting health-behavior change [45]. One of the few studies that investigated the influence of other organizational factors reported a 13% increase in participation in companies with a strong communication strategy [45]. This refers to the extent to which a strategic, comprehensive, integrated communications plan with multiple communications pieces and delivery channels tailored to the employee population is used by companies that offer WHPPs to their work force. Differences in communications strategy during the process of invitation to and inclusion in the HRA could have accounted for some of the variety in participation among the five organizations in the current study. For instance, among the participating organizations in our study, the university medical center had the highest participation (51.7%). In this organization, participation was actively encouraged by upper and middle management, and the HRA was highlighted in the in-house magazine.

By extension, the recruitment message used by organizations can result in selection among participants: whereas Organization A may emphasize one specific feature of the WHPP (eg, “increase your vitality by participating”), Organization B may emphasize another (eg, “screening for health risks”). Following this line of reasoning, the lack of consistent reports in the literature on most individual characteristics of participation may have been caused in part by the widely varying content of recruitment messages. Future research into the reach of WHPPs should consider these and other communication aspects. Based on the combined insight of individual and organizational characteristics of participation, framing the recruitment message could prove to be an essential tool for companies trying to reach employees with specific risk profiles.

### Strengths and Limitations

A limitation of the current study is the low response of the nonparticipants to the nonparticipant questionnaire. Others have been confronted with comparable limitations [36,41]. Individuals who are unwilling to participate in a program are also less likely to respond when asked to participate in a derivative of that program, which in our study was the request to complete a nonparticipant questionnaire. However, in our study, questionnaire responders were of the same age and gender as those who did not respond. Therefore, it is less likely that the reported results have been influenced by selection bias. A strength of the current study is the large size of our study cohort.

No individual characteristics were available for dropouts other than age and gender. This is also a limitation of the current study. Although the number of dropouts (7.9%) was relatively low, their inevitable exclusion from the participant group could have had some influence on the reported findings.

Except for age and gender, which were available from the HRA invitation lists for nearly all (>99.5%) invitees, data on other individual characteristics were collected differently for participants and nonparticipants. For participants, data were collected from the Web-based health questionnaire component of the HRA as part of a larger set of health data collected to generate a personal health report. A separate, short online questionnaire was created to collect data on individual characteristics from the nonparticipants. Some might argue that this divergence in data collection threatens the reliability of the reported findings. However, we estimate this effect to be small, as both participants and nonparticipants completed a set of questions online that were identical with respect to the individual characteristics used in this study.

### Conclusion

This study showed health-related participation in a Web-based HRA. With regard to isolated health behaviors (insufficient physical activity, excess alcohol consumption, and stress), those who could benefit most from the HRA were more likely to participate. Employees who rated their overall health as less than positive and tobacco users were less likely to participate. Web-based delivery of the WHPP did not result in selective participation by more highly educated, female, or older employees.

## Abbreviations

BMI: body mass index
HRA: health risk assessment
WAI: work ability index
WHPP: worksite health promotion program
